# Characterizing clinical heterogeneity in an inpatient service treating mental, substance use and concurrent disorders

**DOI:** 10.1371/journal.pmen.0000074

**Published:** 2024-07-08

**Authors:** Marie N. S. Gendy, Shannon Remers, Jean Costello, Brian Rush, James MacKillop

**Affiliations:** 1 Peter Boris Centre for Addictions Research, McMaster University & St. Joseph’s Healthcare Hamilton, Hamilton, Ontario, Canada; 2 Department of Psychiatry and Behavioral Neurosciences, McMaster University, Hamilton, Ontario, Canada; 3 Homewood Research Institute, Guelph, Ontario, Canada; 4 Michael G. DeGroote Centre for Medicinal Cannabis Research, McMaster University & St. Joseph’s Healthcare Hamilton, Hamilton, Ontario, Canada; 5 Homewood Health Institute, Guelph, Ontario, Canada; 6 Center for Addiction and Mental Health, Toronto, Ontario, Canada; 7 Department of Psychology, Neuroscience, and Behavior, McMaster University, Hamilton, Canada; BronxCare Health System, UNITED STATES

## Abstract

Patients diagnosed with concurrent disorders (CD)—comorbid substance use disorder with other psychiatric conditions—experience poorer clinical outcomes, and significant gaps remain in defining the optimal care path for treating CD. Toward this goal, the primary aim of this study was to characterize individual differences in substance use and psychiatric symptomology in an inpatient clinical sample using a person-centred approach. Admission assessment data from a private inpatient service treating mental disorders, substance use, and concurrent disorders was used (n = 177). Latent profile analysis (LPA) was performed to classify individuals into statistically distinct latent profiles based on their psychiatric symptoms and polysubstance use as covariates. LPA revealed four profiles. Profile 1 (20%) was identified as having low SUD and low psychiatric disorders, profile 2 (65%) was identified as having low SUD and high psychiatric disorders, profile 3 (8%) was characterized as high substance use and moderate psychiatric disorders and profile 4 (7%) was identified as the high SUD and high psychiatric disorders. The participants in the two profiles endorsing high SUDs, Profiles 3 and 4, showed significantly higher impulsivity in terms of higher positive urgency sensation-seeking scores compared to the other profiles and the highest use of cocaine/stimulants than the other two. Identifying clinical heterogeneity by classifying individuals into distinct profiles is a first step toward designing more targeted and personalized interventions in clinically complex inpatient populations.

## Introduction

Compared to the general population, people seeking treatment for a diagnosed mental health condition such as anxiety, depression, attention-deficit hyperactivity disorder (ADHD), bipolar disorder, personality disorders, and schizophrenia are twice as likely to have a co-occurring substance use disorder (SUD) [1]; at least 20% of people with a mental illness have a diagnosis of SUD. Similarly, people with SUD are three times more likely to have a comorbid mental illness; approximately 15% of people with a SUD have a co-occurring mental health condition [1]. The complex mechanisms leading to co-occurring SUD and mental health conditions are putatively explained by genetic, environmental, and, indeed, bidirectional causality [2]. Research has found that people with mental disorders may use alcohol and illicit drugs to self-medicate [3]. Additionally, brain changes occurring in people with mental disorders may enhance the rewarding effects of psychoactive substances, making them more likely to continue using them [3].

These co-occurring disorders, commonly called concurrent disorders (CD) or dual diagnosis, are considered an enormous challenge in mental health and substance use treatment programs [4–6]. Studies have shown that patients diagnosed with CD experience poorer outcomes as they are underdiagnosed and experience a more chronic and treatment-resistant trajectory [6, 7].

People with CD face complex diagnoses, treatment, and increased risks of health complications, social marginalization, legal issues, and stigma [8]. People with CD are at a higher risk of both mortality and morbidity. This risk is primarily due to premature drug-related deaths and an increased risk of suicide [6, 9]. Individuals with CD are often part of a highly vulnerable population with multiple biological, psychological, and social risk factors. As a result, the course of both types of conditions can be more severe and complicated due to persistent risk factors [2, 10]. Furthermore, substance use disorders and non-substance-use mental disorders have an intertwined impact, affecting the course and prognosis of both. This makes the management of concurrent disorders very complex [2, 11]. There are gaps in substance use and mental health treatment. Unmet need is higher for substance use as psychiatrists. Psychiatrists and addiction specialists don’t always collaborate on treatment [12]. Despite the treatment gap, there has been an increase in the utilization of healthcare services. For example, in a Canadian cohort study, individuals with concurrent disorders had significantly higher odds of emergency department use, hospitalization, and primary care visits than those with either substance use disorder or non-substance-related mental disorders [13]. Fundamentally, there remain major gaps in knowledge around the optimal approaches for treating concurrent disorders [14].

Existing models for CD treatment include sequential, parallel, or integrated treatment [15]. The sequential model concept treats one condition followed by another. In contrast, the parallel model concept treats SUD in an addiction service while treating the psychiatric symptoms in a different psychiatric service. On the other hand, the integrated treatment concept combines addiction and other mental health conditions within the same treatment setting [16, 17].

A systematic review has shown that integrated models of care are more effective than conventional, non-integrated models. Integrated models have shown greater effectiveness than standard care models in reducing substance use disorders and improving mental health in patients with concurrent disorders. The review has also indicated that the integrated model is more cost-effective than standard care. Collectively, recent recommendations favor the integrated treatment model as it provides better outcomes [7, 18], but recent studies suggest that the quality of the established integrated treatment programs could nonetheless be improved [12]. A systematic review evaluating CD guidelines over the past 20 years suggests potential improvements, including staging or grading patients or improving the match between patient conditions and treatment programming [12].

One of the challenges for CD treatments is the inherent clinical heterogeneity, given the many possible permutations of conditions. Determination of the appropriate path of care, selecting therapeutic targets, and matching patients to appropriate therapies are all forms of treatment personalization. Personalized treatment, which refers to an individualized approach to tailoring customized treatment plans based on individuals’ clinical variables, is gaining much interest [19, 20]. One strategy is to examine program-level data to identify overall characteristics and, more importantly, observe the typical co-occurring patterns of presenting symptoms. This can be undertaken using statistical approaches for characterizing latent clusters of patients, such as latent profile analysis (LPA). This approach and related techniques are person-centered [21] insofar as the results are driven by person-level constellations, as opposed to mean-centered analyses that distill variability into overall means and measures of variability (e.g., standard deviation, variance) [22]. LPA is a multivariate approach [23] that portrays unobserved correlations among critical variables to configure underlying profiles [24]. Each profile emerges with its unique substance use pattern, psychiatric differences, and behaviors. A clear clinical understanding of the patient’s unique profiles allows the design of personalized treatment plans that cater to their specific needs. By tailoring the approach based on each profile, clinicians can provide more effective care that leads to better treatment outcomes. For example, a profile struggling with high substance use disorder may require a different approach than a profile with a prominent psychiatric disorder. Understanding these differences is crucial in creating targeted interventions that can help patients overcome their challenges.

This statistical technique has been previously used in mental health services to identify different profiles of patients. For instance, LPA was used to determine distinct emerging profiles among individuals who use marijuana in a large sample of college students. Their results revealed different profiles with distinct risk factors suggesting different treatment plans [25]. Further, LPA was used to determine heterogeneity among emerging adults reporting heavy episodic drinking in two samples, one American and one Canadian, showing the same underlying profiles [26]. Moreover, LPA revealed different profiles of patients with varying risks of premature treatment termination to develop strategies for individuals showing high-risk termination [24].

Given these promising findings, the current study’s main goal was to characterize individual differences in psychiatric and substance use symptoms in a concurrent treatment service using a person-centred approach and latent profile analysis. Further, this study focuses on a specific mechanism of behavior change: impulsivity. According to the Diagnostic and Statistical Manual of Mental Disorders, DSM-5, impulsivity is defined as the lack of self-regulatory capacity [27]. High impulsivity is common in various mental disorders, including bipolar and schizophrenia spectrum disorders, attention-deficit hyperactivity disorder, borderline and antisocial personality disorders, and intermittent explosive disorder [28, 29]. Impulsivity has also been linked to violent behaviors and treatment targets for suicidality and aggression (34,35). Regarding SUD, high levels of impulsivity and impairments in self-regulation were found to be reliable predictors of drug-seeking, treatment outcome, remission rates, and relapse behaviors [30–32]. Previous studies have found consistent associations between several impulsivity facets, such as decision-making, cognitive disinhibition, and delay discounting, and poor treatment outcomes among individuals diagnosed with SUDs [33, 34]. Thus, impulsivity could inform differential motivational features for specific profiles. Research suggests that patients with high levels of impulsivity across various psychiatric diagnostic categories may benefit from preventive and therapeutic strategies that specifically target impulsivity [35]. This could potentially improve outcomes and quality of life for these individuals [35]. Therefore, we aimed to identify which emerging profiles exhibit elevated impulsivity and impairments in self-regulation and may benefit from impulsivity-related programming. Specifically, the second objective was to explore how different profiles of patients varied in several facets of impulsivity, namely impulsive personality traits and overvaluation of immediate rewards.

## Methods

### Ethics statement

Data collected via routine clinical assessment were accessed retrospectively via research protocol that received ethics approval from the Regional Center for Excellence, Research Ethics Board in Guelph, Ontario, Canada (REB #22–01). The data was accessed for research analysis in April 2022. The patients completed informed consent at the beginning of the treatment. Patients were informed that their data might be used for research, and they had the option to opt-out to ensure voluntary participation at the time of data collection.

### Participants

This study used the admission data of individuals admitted to Homewood Ravensview (elective private inpatient mental health and SUD services) between (June 2019 –March 2020; n = 177).

Homewood Ravensview provides immediate, expert treatment for people living with mental health, addiction, and concurrent conditions, offering private inpatient mental health services. An integrated treatment is offered through standard treatment modalities, which include CPT, CBT, DBT, motivational interviewing, and delivering evidence-based, medically-led treatment. It is a voluntary program that is self-pay, insurance, or a funding source.

Data were collected electronically as part of routine clinical assessment, using psychometrically evaluated scales that measured a variety of clinically relevant domains. At the time of the study, the program offered a 9-week treatment program treatment for people living with mental health, trauma, addiction, and concurrent conditions. Treatment costs were covered through a variety of sources, including public (e.g., provincial funding), semi-private (e.g., health insurance), or private (e.g., out-of-pocket) funding. No individuals were involuntarily admitted to treatment.

To inform assessment and treatment planning, the patients completed electronic intake self-reported assessments within the first seven days of admission, measuring symptomology associated with mood, anxiety, and substance use.

### Measures

SUD symptoms were evaluated using the patient-endorsed symptoms of the Psychoactive Substance Use Module from the International Classification for Diagnosis (ICD)–10 Symptom Checklist for Mental Disorders [36] with supplemental symptoms of SUD for DSM-5 [37]. The scoring system for SUD symptoms ranges from 0 to 11, with self-reported response options of ’yes’ or ’no’ for each symptom. The checklist includes substances such as cannabis, alcohol, cocaine, opioids, other stimulants, hallucinogens, inhalants, prescription sedatives, prescription sleep aids, and other substances not mentioned in the list. The patient-reported SUD symptoms have been validated previously through a structured clinical interview [38].

### Major Depressive Disorder (MDD)

Depressive symptoms were assessed using the Patient Health Questionnaire (PHQ-9) [39]. It consists of nine questions scored from 0 to 3. We used 16 or above as a cut-off score to indicate whether individuals met the criteria for MDD [38].

### Generalized Anxiety Disorder-7 (GAD-7)

The GAD-7 [40] was used to assess the symptoms of anxiety disorders, a brief, self-report, 7-item measure, in which each item is rated from 0 (not at all) to 3 (nearly every day), yielding a total score out of 21. A cut-off score of 9 or above was used to indicate whether individuals had GAD [38].

### Post-Traumatic Stress Disorder (PTSD)

Symptoms of PTSD were assessed using the PTSD checklist of DSM-5 (PCL-5) [41], a brief, self-report, 20-item measure with items ranging from 0 (not often) to 4 (extremely). A score of 42 or above was used as the cut-off score to indicate whether individuals had probable PTSD [38].

### Impulsivity

The Impulsive Behavior Scale (UPPS-P) and the delayed discounting task (DDT) were used to assess impulsivity. The (UPPS- P) is a self-report measure with four items assessing each of five dimensions of impulsive behavior: negative urgency, positive urgency, lack of perseverance, lack of premeditation, and sensation seeking. Items are rated from “strongly agree” (1) to “strongly disagree” (4). The high scores in positive urgency, negative urgency, and sensation seeking indicate a "highly disagree" response, which translates into low impulsivity [42].

The Delay Discounting Task (DDT) was a behavioral task that presented participants with a choice between smaller-immediate and larger-delayed monetary rewards, reflecting individual impulsivity [43]. In this study, participants were offered two delayed rewards of either $100 or $1,000 and had to choose between a constant delayed amount and half that amount available immediately. Based on their response patterns, the hyperbolic temporal discounting rate (*k*) was calculated. A higher *k* value indicates that individual discounts prefer smaller immediate rewards compared to larger future rewards.

59.3% of the sample was males, mostly of white race (84%), and the mean (SD) age was 41.7 (13.8). The percentage of participants who screened positive for depression, anxiety, and PTSD was (61.6%, 84.2%, and 67%, respectively). The percentage of participants who screened positive for alcohol, cannabis, cocaine, and stimulants were endorsed by (44.1%, 19.8%, 13.6%, and 6.8%, respectively). Other substances were less endorsed, (Table 1).

**Table 1:** Participant characteristics (N = 177).

Demographics	
**Age** *Mean* (SD)	41.7 (13.8)
**Sex**	(%)
**Male**	59.3
**Race**	(%)
**Caucasian**	84
**Asian**	4.5
**1**^**st**^ **nation**	6.2
**Pacific/Latin**	0.6
**Mixed**	4.5
**Marital Status**	(%)
**Married**	20.3
**Psychiatric Disorders** (*Mean* (SD)/% screening positive)*	
**Depression: PHQ-9**	17 (6)/ 61.6%
**Anxiety: GAD-7**	14.2 (5.4)/ 84.2%
**PTSD: PCL-5**	49.3 (17.5)/ 67.8%
**SUD status** (% screening positive)^+^	
**Alcohol**	44.1
**Cannabis**	19.8
**Cocaine**	13.6
**Stimulants**	6.8
**Opioids**	4.5
**Sleep Aids**	5.1
**Sedatives**	5.6
**Hallucinogens**	2.8
**Other Drugs (not listed above)**	2.8

*: Percentage of the participants who screened positive for each psychiatric condition.

_+_: endorsing two or more symptoms of the DSM-5 SUD criteria.

### Data analysis

Descriptive analyses (means, standard deviations, percentages) were conducted to describe patient demographic characteristics. Latent profile analysis was performed to classify patients into statistically distinct latent profiles based on patterns of responses indicating SUD and co-occurring psychiatric symptoms (i.e., Alcohol, Cannabis, Cocaine/Stimulants, other substances, PHQ-9 for depression, GAD-7 for anxiety and PCL-5 for PTSD). The *MPlus* maximum likelihood robust estimation was used in all models [44]. To determine an optimal profile solution, each model was estimated by sequentially adding profiles and testing for the best model fit. The Akaike Information Criterion (AIC), Bayesian Information Criterion (BIC), sample size adjusted BIC, Lo-Mendell-Rubin (LMR) test, and entropy were used as indicators to assess the best model fit for an optimal profile solution. Lower values of AIC and BIC and adjusted BIC indicated superior fit. The LMR p-value compares the current profile, k, to the previous profile, k-1 improvement in fit between profile models. An entropy value approaching 1 indicates a better model fit and is used as an index for better model classification. The assignment of the probability of the optimal profile solution profile was assessed to verify the precision of group classification and membership probability. Once an optimal profile solution was selected, each profile’s demographic and clinical characteristics were compared. Profile-specific distal variables (age and sex were used as predictors, and impulsivity measures (UPPS and DDT scores) were used as outcomes for each profile) were examined using the *MPlus* 3 steps model and Wald’s Chi-square χ^2^ difference test [45]. In addition, the chi-square statistic was converted to Cohen’s *d* as a measure of effect size [46]. Means comparisons were performed with a one-way between-subjects ANOVA test. A sample *t*-test was performed to compare continuous variables across patients, and the Pearson χ^2^testχ^2^ was used for categorical variables. For all analyses, statistical significance was set at *p* < 0.05. Data analyses were performed using MPlus v.8.6.

## Results

### Latent profile analysis

**Fig 1:**
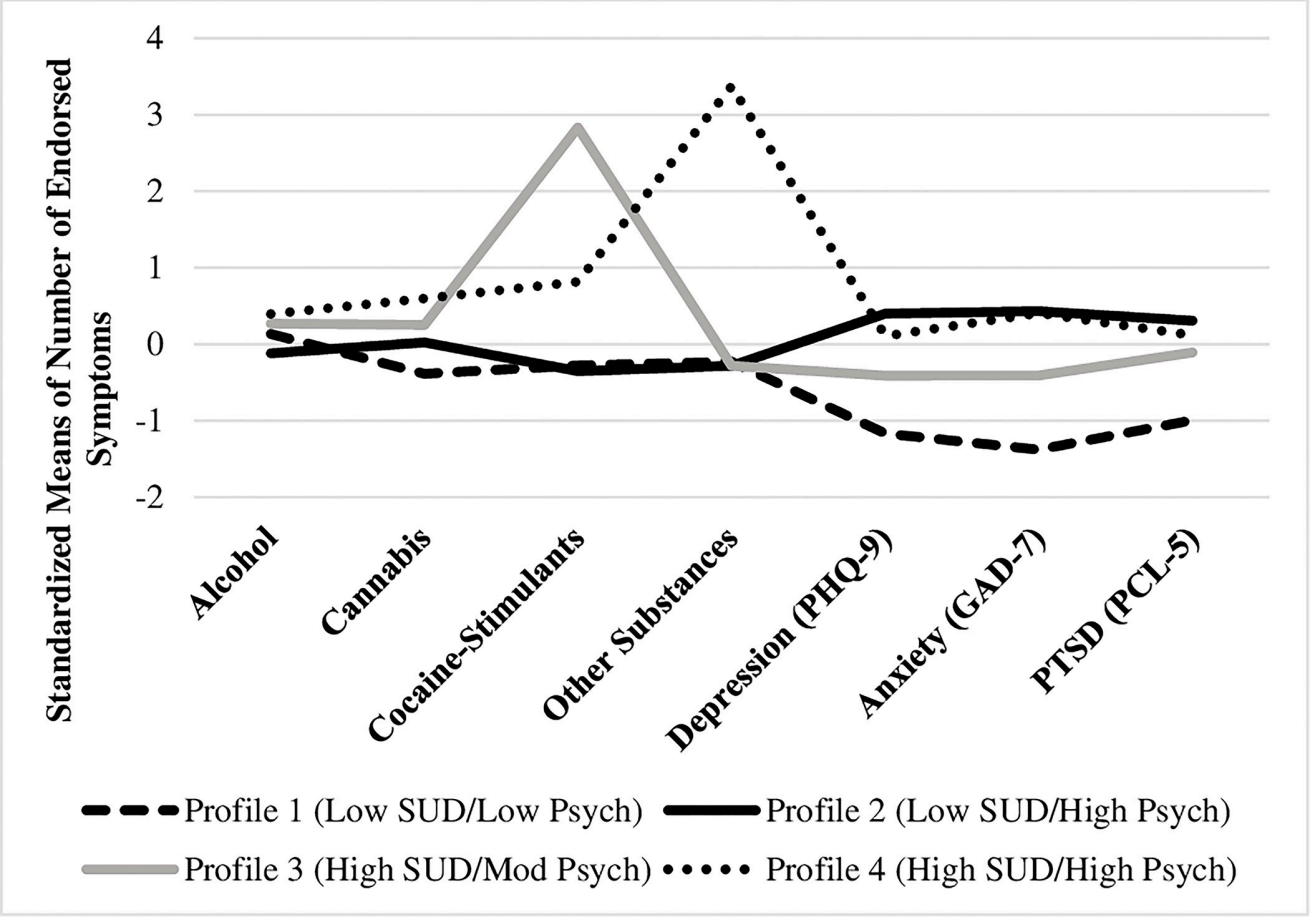
Estimated standardized means of latent profile indicators for the four-profile solution.

LPA analysis revealed that the four-profile solution was the best fit due to the following reasons: (a) lower AIC and BIC relative to the other profile solutions, (b) the highest entropy value, (c) LMR- p-value was significant through the four profile-model (p < 0.05) and reached no significance when the model was expanded to a 5 profile solution (Table 2). Therefore, four distinct profiles emerged, as shown in Fig 1. The average latent profile probabilities for the most likely profile membership are shown in Table 2. Probabilities were approaching 1.0, which indicates their high fit probability.

**Table 2:** Model fit statistics across latent profile solutions and average latent profile posterior probabilities.

**Number of Profiles**	**2 Profiles**	**3 Profiles**	**4 Profiles**	**5 Profiles**
**AIC**	3213.79	3067.51	2938.73	2873.98
**BIC**	3283.66	3162.79	3059.42	3020.08
**Adjustedusted BIC**	3213.99	3067.79	2939.08	2874.41
**Entropy**	0.99	0.91	0.93	0.93
**LMR**	338.17	158.45	136.85	78.85
**LMR- P value**	0.09	0.01	0.002	0.18
**Average latent profile posterior probabilities for most likely latent profile membership N (Row) by latent profile (column)**				
**Profiles**	**1**	**2**	**3**	**4**
**N1**	0.914	0.086	0	0
**N2**	0.03	0.968	0	0
**N3**	0	0.006	0.994	0
**N4**	0	0	0	1

The proportion of participants in each profile was as follows: Profile 1 (20%) was identified as having overall low severity concerning substance use and psychiatric comorbidities *(Low SUD/Low Psych)*, profile 2 (65%) was identified as having high psychiatric conditions with low SUD severity *(Low SUD/High Psych)*, profile 3 (8%) was characterized by high substance use, especially stimulants, and moderate psychiatric symptoms *(High SUD/Mod*. *Psych)*, profile 4 (7%) was identified as the high SUD and high psychiatric comorbidities *(High SUD/High Psych)*.

### Demographic and clinical differences by latent profile

All characteristics and differences of the four profiles are presented in detail in Table 3. Age was a significant predictor for classification. In the *(Low SUD/Low Psych)* profile and the *(Low SUD/High Psych)* profile, the participants were significantly older [*Mean* (SD) = 48.97 (14.27), 41.97 (13.31), respectively] compared to the other profiles (p < 0.001).

**Table 3:** Characteristics of the four profiles.

**Mean (SD) N (%)**	**Profile 1 (Low SUD/Low Psych) (20%)**	**Profile 2 (Low SUD/High Psych) (65%)**	**Profile 3 (High SUD/Mod Psych) (8%)**	**Profile 4 (High SUD/High Psych) (7%)**	**Statistical Significance *P***
**Demographics**					
**Age** *Mean* (SD)	48.97 (14.27)	41.97 (13.31)	29.78 (4.37)	32.15 (10.22)	< 0.001
**Sex** (%)					0.04
**M**	48.6	61.7	71.4	53.8	
**Psychiatric Disorders (***Mean* (SD)/% screened)/%)*) *					
**Depression: PHQ-9**	9.86 (4.74)/14.28	19.29 (4.51)/76.52	14.50 (6.09)/50.00	17.54 (4.13)/69.23	< 0.001
**Anxiety: GAD-7**	6.5 (3.8)/34.28	16.6 (3.1)/100.00	12.1 (5.4)/64.28	16.5 (4.7)/100.00	< 0.001
**PTSD: PCL-5**	31.6 (13.3)/22.86	54.7 (15.2)/80.87	47.6 (19.3)/12.00	51.4 (14.7)/76.92	< 0.001
**SUD Endorsement** (% screening positive)^+^*					
**Alcohol**	3.65 (3.61)/ 60.00	2.55 (3.59)/37.39	4.07 (4.65)/50.00	4.53 (4.61)/53.84	0.11
**Cannabis**	0.26 (0.88)/2.86	1.23 (2.47)/20.87	1.78 (2.66)/28.57	2.61 (3.30)/46.15	0.01
**Cocaine-Stimulants**	0.37 (1.16)/8.57	0.14 (0.67)/4.35	9.57(1.87)/100.00	3.61 (4.11)/69.23	< 0.001
**Other Substances** ^**##**^	0.26 (0.70)/ 8.57	0.16 (0.66)/3.48	0.14 (0.53)/7.14	9.38 (1.76)/100.00	< 0.001

#: Other substances include (opioids, hallucinogens, inhalants, sedatives, sleep aids, and substances not listed).

*: Percentage of the participants who screened positive for each psychiatric condition.

+: endorsing two or more symptoms of the DSM-5 SUD criteria.

Sex was another distinct predictor for classification, and there were significant differences between profiles. The (High SUD/Mod. Psych) profile had a higher male ***representation*** than the *(Low SUD/Low Psych)* profile (71.4% and 48.6%, respectively, p = 0.04).

**Fig 2:**
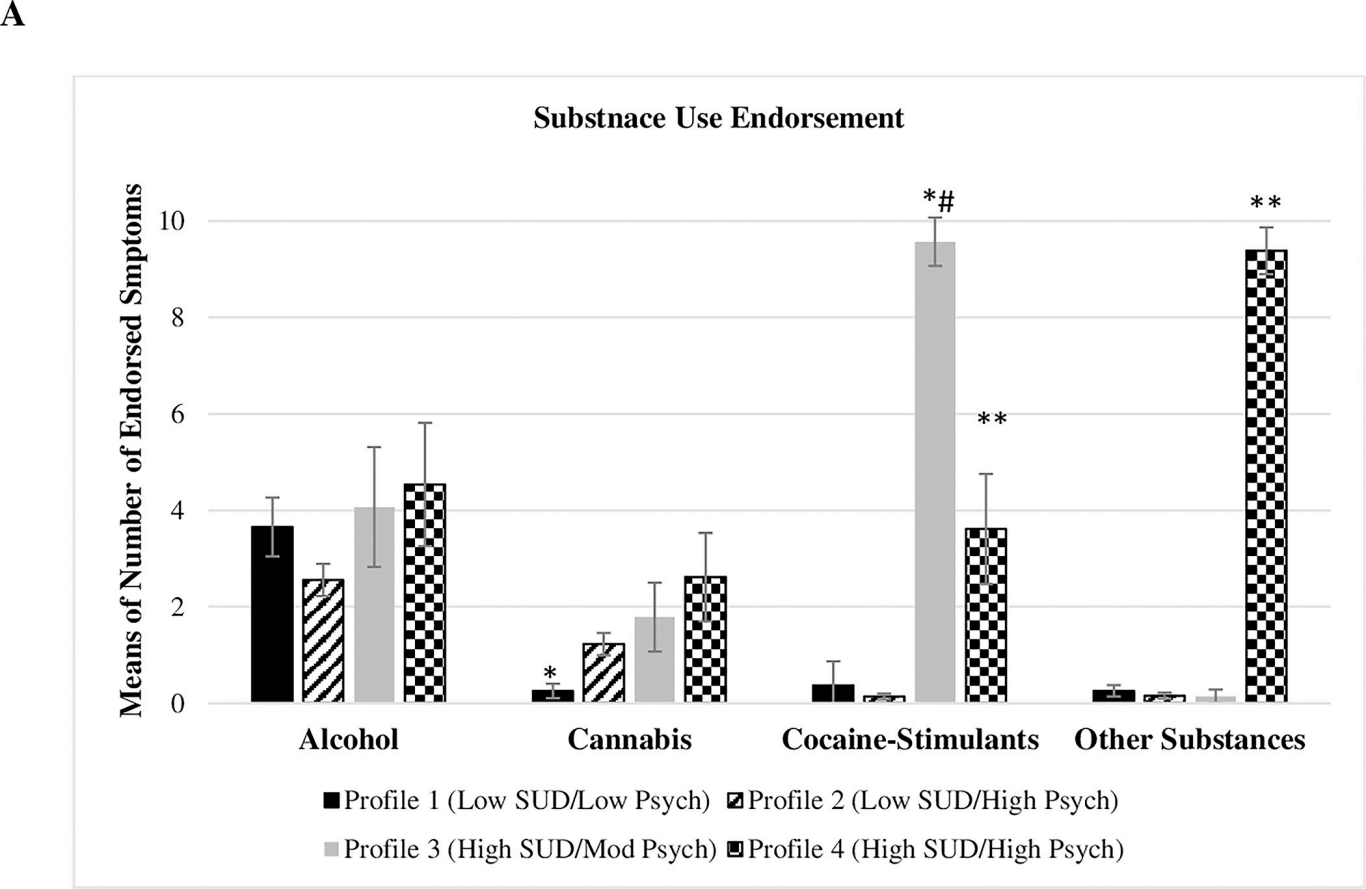
Latent profile differences in substance use disorder and psychiatric comorbidities. Panel A represents latent profile differences in terms of substance use endorsement. Panel B represents latent profile differences in anxiety and depression score means. Panel C represents latent profile differences in terms of PTSD score. Dashed lines represent the cut-off scores for anxiety (9), depression (16), and PTSD (42). *: Profile 1 is significantly different from the other profiles. *#: Profile 3 is significantly different from the other profiles.

Substance endorsement details of the four profiles are shown in Table 3 and Fig 2. Alcohol endorsement was present in all four profiles with no significant mean difference [F (3,173) = 1.98, p = 0.11]. Cocaine/Stimulants were highly endorsed in *(High SUD/Mod*. *Psych)* profile, where 100% of the patients screened positive for cocaine-stimulants [*Mean* (SD) = 9.57 (1.87)], followed by the *(High SUD/High Psych)* profile [69.2% of the patients screened positive, *mean* (SD) = 3.61 (4.11)] compared to the other profiles, [F (3,173) = 201.4747, p < 0.001, *d* > 1)]. Cannabis use was positive in 46.2% of the patients in the *(High SUD/High Psych)* profile [*Mean* (SD) = 2.61 (3.30)], compared to the *(Low SUD/Low Psych)* profile [*Mean* (SD) = 0.26 (0.88), F (3,173) = 3.77, p = 0.01, *d* > 1]. The polysubstance use was highly endorsed in the *(High SUD/High Psych)* profile compared to the other profiles as shown by comparing the means, [100% of the patients screened positive, *Mean* (SD) = 9.38 (1.76), F (3,173) = 552.3, p < 0.001, *d* > 1].

The details of the four profiles for psychiatric comorbidities are shown in Table 3 and Fig 2. Depression scores were significantly low in the *(Low SUD/Low Psych)* profile [14.3% screened positive, *Mean* (SD) = 9.9 (4.7)] compared to the *(Low SUD/High Psych)* profile [76.5% screened positive, *Mean* (SD) = 19.28 (4.51)] and the *(High SUD/High Psych)* profile [69.2% screened positive, *Mean* (SD) = 17.54 (4.13), F (3,173) = 37.82, (p < 0.001), *d* = 0.7]. 100% of the patients in both the *(Low SUD/High Psych)* the *(High SUD/High Psych)* profiles screened positive for anxiety [*Mean* (SD) = 16.64 (3.14) and 16.46 (4.75), respectively] compared to the *(Low SUD/Low Psych)* profile [34% screened positive, *Mean* (SD) = 6.48 (3.84), *d* = 0.7 and 0.6], and the *(High SUD/Mod Psych)* profile [64.3% screened positive, *Mean* (SD) = 12.07 (5.48), F (3,173) = 72.95, p < 0.001). PTSD scores were significantly low in the *(Low SUD/Low Psych)* profile compared to the other profiles [F (3,173) = 21.01, p < 0.001].

### Latent profile differences in impulsivity measures

**Fig 3:**
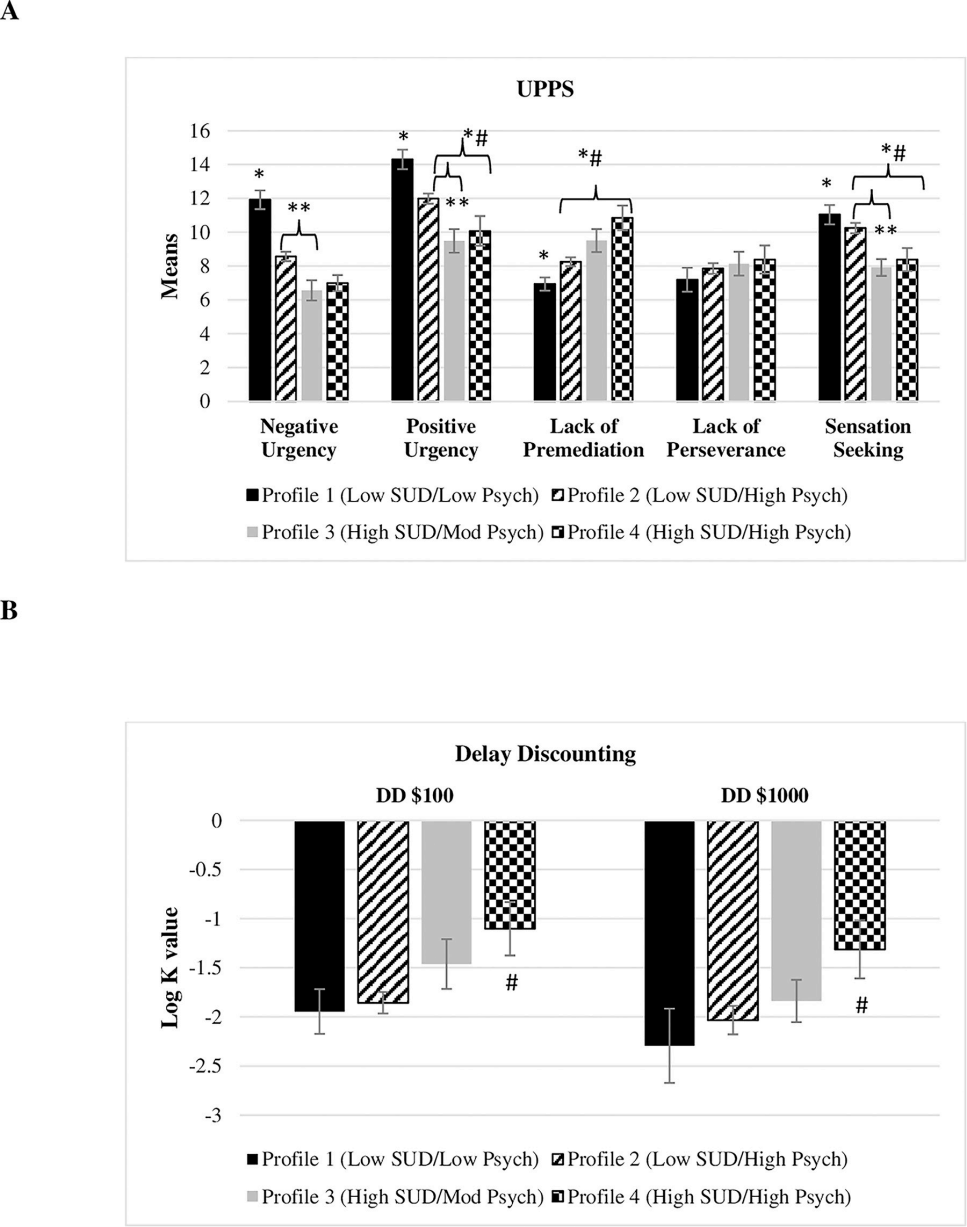
Latent profile differences in impulsivity. Panel A represents latent profile differences in terms of UPPS facets, and Panel B represents latent profile differences in terms of the Delay discounting test (DDT). A: *: Profile 1 is significantly different from the other profiles. **: Profile 2 is significantly different from profile 3. *#: Profile 2 is significantly different from profile 4. B: #: Profile 4 significantly differs from profiles 1 and 2. This test used two delayed rewards of either $100 or $1,000.

Overall, participants in the *(High SUD/Mod Psych)* and the *(High SUD/High Psych)* profiles showed the highest impulsivity domains compared to the other two profiles (*χ*^*2*^ = 59.13, p < 0.001). Similarly, participants in the *(High SUD/Mod Psych)* and the *(High SUD/High Psych)* profiles showed significantly higher positive urgency compared to the other profiles (*χ*^*2*^ = 34.41, p < 0.001). Regarding lack of premeditation, participants in the *(High SUD/Mod Psych)* showed a higher score compared to the *(Low SUD/Low Psych)* profile (*χ*^*2*^ = 10.69, p = 0.001). At the same time, participants in the *(High SUD/High Psych)* profile showed a higher lack of premeditation compared to the *(Low SUD/Low Psych)* and the *(Low SUD/High Psych)* profiles (*χ*^*2*^ = 22.25, p < 0.001 and *χ*^*2*^ = 11.16, p = 0.001).]. Participants in the *(High SUD/Mod Psych)* and the *(High SUD/High Psych)* profiles showed significantly higher sensation seeking compared to the other profiles (*χ*^*2*^ = 26.24, p < 0.001). No significance was detected between the four profiles for the lack of perseverance (Fig 3). For a moderate delayed reward ($100) and large delayed reward ($1000), participants in the *(High SUD/High Psych)* profile had significantly higher Log_10_ *k* values compared to the *(Low SUD/Low Psych)* profile; (*χ*^*2*^ = 5.62, p = 0.02 and *χ*^*2*^ = 4.20, p = 0.04) and the (Low SUD/*High Psych)* profile; (*χ*^*2*^ = 6.63, p = 0.01 and *χ*^*2*^ = 4.79, p = 0.03) (Fig 3).

## Discussion

Given the need to improve integrated treatment programs targeting individuals diagnosed with SUD and co-occurring mental health conditions [12] and, considering the inherent clinical heterogeneity in concurrent disorders [12], one potential way to improve is to understand the nature of the heterogeneity and customize care paths idiosyncratic programs. Specifically, classifying heterogeneity among patients into distinct profiles is a promising strategy to allow clinicians to design more targeted and personalized interventions accordingly. Toward this end, the main objective of this study was to identify different patterns of heterogeneity in terms of substance use and psychiatric disorders among individuals seeking addiction treatment. To achieve this, a person-centered latent profile analysis approach was applied to classify individuals into different profiles based on their unique characteristics. The study aimed to determine which profile exhibits high levels of impulsivity and self-regulation difficulties, as these traits are known to increase the likelihood of drug-seeking behavior and relapse. The results of this study may help clinicians develop more effective profile-based treatment programs for individuals struggling with concurrent disorders.

To that end, the results showed the emergence of four distinct profiles that differ in their demographics, SUD, psychiatric disorders, and levels of self-regulation. Profile 1 was identified as having overall low substance use and low psychiatric comorbidities *(Low SUD/Low Psych)*, profile 2 was characterized by having low SUD endorsement and high psychiatric conditions *(Low SUD/High Psych)*, profile 3 was a high substance of use profile and moderate psychiatric symptoms *(High SUD/Mod Psych)*, and profile 4 was the high SUD and high psychiatric comorbidities *(High SUD/High Psych)*. These findings are broadly consistent with previous studies using LPA to identify different profiles in addiction programs. An inpatient SUD treatment program found four latent profiles: high alcohol/low psychiatric severity, low alcohol use/ high levels of drug use, and highest levels of psychopathology, a third profile with the highest levels of alcohol use/lowest levels of drug use, and high levels of psychopathology, and a fourth profile with the lowest levels of alcohol use, high levels of drug use, and low levels of psychopathology [24]. The high psychiatric symptoms with low drug use and the high drug use with low psychiatric comorbidities profiles are consistent with our profiles (Low SUD/High Psych) and (High SUD/Mod Psych) findings. Another LPA of inpatients showed a high alcohol severity/low psychiatric severity, a high drug/psychiatric severity, a high alcohol/psychiatric severity, and a high drug severity/low psychiatric severity [47]. Similarly, the high drug severity/low psychiatric severity is comparable to the *(High Sud/Mod Psych)* profile. In contrast, in an outpatient SUD treatment setting, LPA revealed four profiles: moderate psychiatric symptoms with moderate polysubstance use and high cannabis use; moderate psychiatric symptoms, high psychiatric profile with moderate cannabis use; and high psychiatric with high polysubstance use profiles [48].

Differences in findings are also clear across studies. Different sample sizes, clinical settings, classification variables, and covariates could explain the differences in profiling. Also, each study was in a different setting, inpatient vs outpatient. What emerges across these studies is clear evidence of latent subgroups of similar patients but different patterns in the clinical setting, suggesting this approach is best suited to be applied on a setting-by-setting basis. Our study was conducted in an inpatient concurrent disorder program. Therefore, we decided to include both SUDs and psychiatric symptoms (depression, anxiety, PTSD) as variables, but individual settings should ultimately determine which variables are most important for classification. Classification.

Age was a significant predictor for classification, as young age was associated with the *(High SUD/Mod Psych)* profile. This finding aligns with research showing that people of a younger age are more likely to endorse different substances of use [49]. Sex was another distinct predictor for classification as it showed significant differences between profiles where the *(High SUD/Mod Psych)* profile showed a higher male representation than the *(Low SUD/Low Psych)* profile. This finding aligns with previous studies, such as a systematic review of latent profiles among young adults that showed that a higher percentage of males consistently predicted membership in the "heavy use" or the "polysubstance use" profile across the studies [50]. Another US study using LPA among subjects with AUD showed that a higher percentage of men was associated with the highest risk profile, and the same profile was the least likely to seek treatment [51].

The results in terms of UPPS and DDT scores of impulsivity showed that the participants in the two profiles endorsing high SUDs showed significantly higher impulsive behavior than the others, consistent with the broader literature [52, 53]. The same two profiles showed the highest use of cocaine/stimulant use. Previous studies also showed highly impulsive behavior associated with profiles endorsing high cocaine use. For instance, a Spanish study of more than 4000 inpatient subjects with SUD found four profiles, and the profile with polydrug use, including cocaine, was associated with higher impulsivity [54]. Another study of outpatient subjects with alcohol and cocaine use disorder showed three profiles based on salience, emotionality, and decision-making determined by DDT. The high impulsivity profile was associated with a high relapse to cocaine and alcohol-cocaine co-use [55]. Our findings that impulsive behavior was high in the polysubstance profiles were consistent with Minhas and colleagues, who found that among the four profiles, the high drug/psychiatric severity showed the highest impulsivity [47]. Developing targeted therapeutic plans for addressing the overvaluation of immediate rewards and impulsive personality traits has considerable promise for improving clinical outcomes across SUDs and a wide range of clinical disorders [56].

The current study has both strengths and limitations. One apparent strength is using a multivariate person-centred statistical approach [23] that allows the characterization of distinct profiles of patients with different degrees of SUD and psychiatric symptoms. Using LPA enabled the emergence of patterns from the observed data based on specific characteristics without making assumptions or focusing on the "average patient." Another strength is that this study was completed in a ‘real world’ treatment setting, increasing ecological validity. However, this study also has some limitations; for instance, all the assessments were patient-reported outcomes that might be subjected to recall bias in some cases. In addition, there was a lack of collateral biomarkers or behavioral tests to verify many of these responses. Another limitation is that this study sample was drawn from a single site, limiting generalizability. Finally, this study was conducted in a semi-private treatment facility and thus may reflect a higher income subset of the population with insurance and may differ from not-for-profit or publicly-funded programs. Further, our study did not account for the presence of personality disorders, such as borderline personality disorder, which isis known to be highly prevalent in individuals with SUDs [57]. Previous research showed that inhibitory control and discounting of rewards, two main facets of impulsivity, are both independently associated with personality disorder. Moreover, the tendency to choose small immediate rewards over larger delayed rewards was associated with borderline personality disorder [58]. Future research should take into consideration other relevant disorders to have more clinically informed profiles.

## Conclusion

There are currently limitations in treating SUDs with comorbid psychiatric symptoms, also known as concurrent disorders. The challenge in treating these patients is their heterogeneity [59]. Therefore, it is essential to characterize clinical heterogeneity systematically so clinicians and programs can create individualized treatment plans and care paths to improve treatment outcomes. The current study identified four distinct profiles having unique patterns of substance use, psychiatric comorbidities, and impulsivity levels. The profiles characterized by high SUD endorsement showed significantly higher impulsive behavior than the others. The same profiles showed the highest use of cocaine/stimulant use. Developing tailored treatment care paths that address these subgroups of patients may optimize outcomes, and testing this hypothesis empirically is a priority for future clinical research on concurrent disorders.

## Supporting information

S1 Data Data file. (XLSX)
